# Expression and Role of Response Regulating, Biosynthetic and Degrading Genes for Cytokinin Signaling during Clubroot Disease Development

**DOI:** 10.3390/ijms21113896

**Published:** 2020-05-29

**Authors:** Rawnak Laila, Arif Hasan Khan Robin, Jong-In Park, Gopal Saha, Hoy-Taek Kim, Md. Abdul Kayum, Ill-Sup Nou

**Affiliations:** 1 Department of Horticulture, Sunchon National University, Suncheon 57922, Korea; rawnak@bau.edu.bd (R.L.); gpb21bau@bau.edu.bd (A.H.K.R.); jipark@sunchon.ac.kr (J.-I.P.); gopalagr@pstu.ac.bd (G.S.); htkim@sunchon.ac.kr (H.-T.K.); kayumagb@pstu.ac.bd (M.A.K.); 2 Department of Genetics and Plant Breeding, Bangladesh Agricultural University, Mymensingh 2202, Bangladesh; 3 Department of Agronomy, Patuakhali Science and Technology University, Patuakhali 8602, Bangladesh; 4 Department of Agricultural Botany, Patuakhali Science and Technology University, Patuakhali 8602, Bangladesh

**Keywords:** Chinese cabbage, cytokinin, clubroot, *Plasmodiophora brassicae*, expression analysis

## Abstract

The obligate biotroph *Plasmodiophora brassicae* causes clubroot disease in oilseeds and vegetables of the Brassicaceae family, and cytokinins play a vital role in clubroot formation. In this study, we examined the expression patterns of 17 cytokinin-related genes involved in the biosynthesis, signaling, and degradation in Chinese cabbage inoculated with the Korean pathotype group 4 isolate of *P. brassicae*, Seosan. This isolate produced the most severe clubroot symptoms in Chinese cabbage cultivar “Bullam-3-ho” compared to three other Korean geographical isolates investigated. *BrIPT1*, a cytokinin biosynthesis gene, was induced on Day 1 and Day 28 in infected root tissues and the upregulation of this biosynthetic gene coincided with the higher expression of the response regulators *BrRR1*, on both Days and *BrRR6* on Day 1 and 3. *BrRR3* and *4* genes were also induced during gall enlargement on Day 35 in leaf tissues. The *BrRR4* gene, which positively interact with phytochrome B, was consistently induced in leaf tissues on Day 1, 3, and 14 in the inoculated plants. The cytokinin degrading gene *BrCKX3-6* were induced on Day 14, before gall initiation. *BrCKX2,3,6* were induced until Day 28 and their expression was downregulated on Day 35. This insight improves our current understanding of the role of cytokinin signaling genes in clubroot disease development.

## 1. Introduction

Clubroot is a devastating disease of *Brassica* vegetables and oilseed crops. This infectious disease is caused by the obligate parasite, *Plasmodiophora brassicae* Woronin. This pathogen is a Cercozoan biotrophic protist of Plasmodiophoraceae family. Clubroot disease is a serious problem of oilseed rape growers throughout the world [1]. The pathogen is variable among geographic regions and therefore regional difference in severity of this disease is reported [2]. According to pathogenic reaction of 12 geographical isolates and the reactions of those isolate to Williams’ differential set the Korean *P. brassicae* isolates were divided into four pathotypes [2,3]. In addition, the intron 1 of 18S smaller subunit of those isolates were found variable after ribosomal DNA sequencing [4].

**Figure 1:**
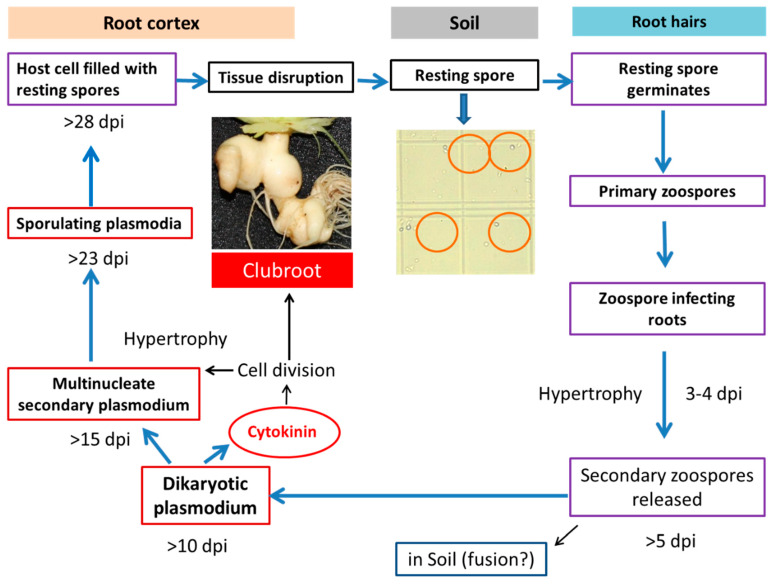
Involvement of cytokinin in clubroot formation during the life cycle of *Plasmodiophora brassicae* (Robin et al. [13] after Dekhuijzen [14], Müller and Hilgenberg [15]). dpi, days post inoculation.

The life cycle of *P. brassicae* is complex (Figure 1) [5,6]. This pathogen is difficult to control because of the durability of spores and lack of suitable chemical control [1,7,8]. Despite the complex life cycle, two distinct phases of infection to its *Brassica* hosts are noted, primary and secondary phases of infection [9,10]. The way of infection and types of roots that are infected differ between primary and secondary phases of infection [11,12]. Primary infection starts from the root hairs and this phase usually lasts first three days (Figure 1). In this phase, the resting spores residing in soil germinates, infects root hairs and releases secondary zoospores. In the secondary phase of infection, the pathogen enters into the cortex of the roots and steles of roots and hypocotyls of the infected plants. When the secondary infection process continues root tissues divide abnormally and that results in rapid swelling in the root zone which is termed as “galls” or “clubs” (Figure 1) [9,11].

It is generally believed that an alteration in the usual hormonal balance has a vital role in the clubroot formation [13,16,17]. The contribution of hormones in club formation in the infected and hypertrophied roots during the invasion of *P. brassicae* was of an area of interest of the many clubroot researchers over the past several decades [14,16,17,18]. Both auxins and cytokinins have key regulatory role at the beginning of hypertrophy in the infected root tissues at the early secondary phase of infection [16,17,19,20,21].

Hormonal homeostasis of auxin and cytokinin is pivotal in the formation of clubroot [21,22,23,24,25]. Stimulation of the cell division which is associated with club formation in the infected roots requires the activity of cytokinin and auxin. In *Arabidopsis thaliana* and *Brassica spp.* an increased level of cytokinin and faster cell division were observed [16,17,26]. Further, cytokinin increases availability of nutrients including carbohydrates, amino acids, and lipids which are essential for the multiplication of pathogen during gall formation [22,26]. At the later stage of club development, a decreasing level of cytokinin primarily reflected a repression of cytokinin biosynthesis genes although few response regulator and receptor genes were induced [16,26,27,28].

Despite the well-recognized importance of auxins and cytokinins in gall formation, the fluctuation in plant hormone contents does not correspond to the severity of infection in *B. rapa* roots [23]. Transcriptome analysis revealed a wide range of variation in the expression of cytokinin biosynthesis-related genes between infected and non-infected tissues. Laser micro-dissection and pressure catapulting (LMPC) detected up-regulated genes associated with cytokinin metabolism in *A. thaliana* during gall formation [29]. Microarray analysis during early phase of root infection at 4, 7, and 10 days revealed significant alteration in expression of a relatively lower number of genes in *A. thaliana* [30]. A few of those genes were found to change the level of expression during infection of the cortex from day 4 to day 10 [30]. By contrast, transcriptomes at two time-points, 10 and 23 days after inoculation, in the root cortex revealed that more than 1000 genes were differentially expressed between infected and control roots [28]. RNAseq data reported 2089 differentially expressed genes in resistant Chinese cabbage line at 30 days post inoculation compared to susceptible line where 188 and 138 genes were associated with plant-pathogen interaction and hormone signal transduction, respectively [24]. In another study, isobaric tags for relative and absolute quantitation (iTRAQ) analysis detected a total of 5003 differentially expressed proteins in resistant vs. susceptible line in secondary phase of disease infection [31].

Malinowski et al. [16] reported that the genes associated with cytokinin biosynthesis, signaling, and degradation show a remarkable repression in host during gall initiation at the secondary phase of infection in *A. thaliana* at 16 and 26 days post inoculation. However, to date, little is known about the behavior of cytokinin regulating, synthesizing, and degrading genes in plants during both primary and secondary phase of clubroot infection and gall expansion in Chinese cabbage. In the current study, we have characterized cytokinin regulating, synthesizing, and degrading genes from seven gene families in silico. We, then, analyzed the expression of 17 cytokinin-related genes, selected based on Schuller et al. [29] in *Arabidopsis thaliana,* in a Chinese cabbage cultivar inoculated with a highly virulent Korean *P. brassicae* isolate during both primary and secondary phase of clubroot infection. However, the specific role of *BrCRR1*, involved in protein phosphorylation, and *BrPYL1*, a receptor of abscisic acid (ABA), in cytokinin accumulation is not obvious. The pattern of expression of cytokinin-related genes was discussed to explain their role during primary infection, gall initiation, and gall expansion. We have also explored an association between increased expression of cytokinin biosynthesis genes and increase/decrease of cytokinin contents (in the published data) at the adjacent time-points in the clubroot infected root tissues to discern the role of cytokinin-related genes. The results of this study shed light on the changes in transcript levels for cytokinin-related genes, both in primary and secondary phases of clubroot infection, involved in metabolism, transport, and signaling in the host, during infection by this important pathogen in Chinese cabbage.

## 2. Results

### 2.1. Disease Severity Index of Four Korean Field Isolates

**Figure 2:**
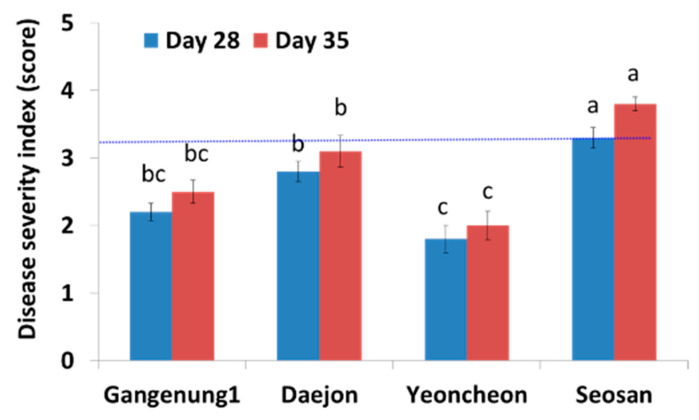
Mean score indicating the severity of clubroot infection in Chinese cabbage cultivar “Bullam-3-ho” at 28 and 35 days after inoculation (DAI) in response to infection with four different Korean *P. brassicae* isolates. Data represent the average disease scores of 10 plants ± standard error. Values with different letters are significantly different at 5% level of significance according to Tukey’s pairwise comparisons.

A small but visible gall appeared at 21 DAI onwards. The isolate Seosan produced the largest galls at both 28 and 35 DAI, followed by Daejon, Gangneung1, and Yeoncheon (Figure 2). Disease severity index (DSI), significantly varied among the four isolates (Figure 2).

### 2.2. Properties of Cytokinin-Related Proteins

The 17 selected cytokinin-related genes were from seven different families: cytokinin oxidase, response regulator, histidine kinase, protein kinase, purine permease, transcriptional adaptor, isopentenyl transferase, and cytokinin-specific binding protein. The isoelectric point of purine permease, transcriptional adaptor, and isopentenyl transferase proteins are >7.0, whereas those of the other protein families are generally <7.0 (Table 1). The molecular weights of these proteins range from 18.37 to 115 kD. Histidine kinase has the highest molecular weight (115 kD). All proteins except isopentenyl transferase, kinase, and cytokinin oxidase were predicted to localize to the nucleus.

### 2.3. Expression Level Difference of Cytokinin-Related Genes in Leaf vs. Root

**Figure 3:**
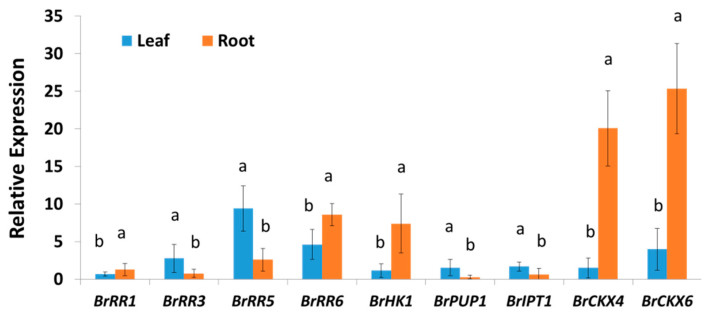
Variation in relative expression of cytokinin regulating, synthesizing, and degrading genes in root and leaf samples. Each data is the average of both inoculated and mock-treated samples at five different time-points (average of 30 samples). Vertical bars represent standard deviations. Letters a and b indicate statistically significant difference between leaf and root samples at 5% level of significance. Gene expression levels in leaves from non-treated plants on Day 0 were set to 1.

Nine cytokinin regulating, synthesizing, and degrading genes exhibited significant variation in their relative expression levels between leaf and root samples as the average of both inoculated and mock-treated samples at five different time-points (Figure 3). Another eight genes showed non-significant variation between leaves and roots (Table S1). Two cytokinin degrading genes, *BrCKX4* and *BrCKX6* showed 13.3- and 6.4-folds higher expression, respectively, in root tissues compared to leaf tissues (Figure 3). Both of the two response regulating genes, *BrRR1* and *BrRR6* accounted for 1.9-fold higher expression in root tissues compared to leaf tissues (Figure 3). By contrast, both *BrRR3* and *BrRR5* genes showed 3.6-fold lower expression in root tissues compared to leaf tissues (Figure 3). Similarly, *BrPUP1* and *BrIPT1* exhibited 5.5- and 2.7-folds lower expression, respectively, in root tissues compared to leaf tissues (Figure 3). But the *BrHK1* gene showed 6.5-fold higher expression in root tissues compared to leaf tissues (Figure 3).

### 2.4. Expression Profiles of Response Regulator Genes

**Figure 4:**
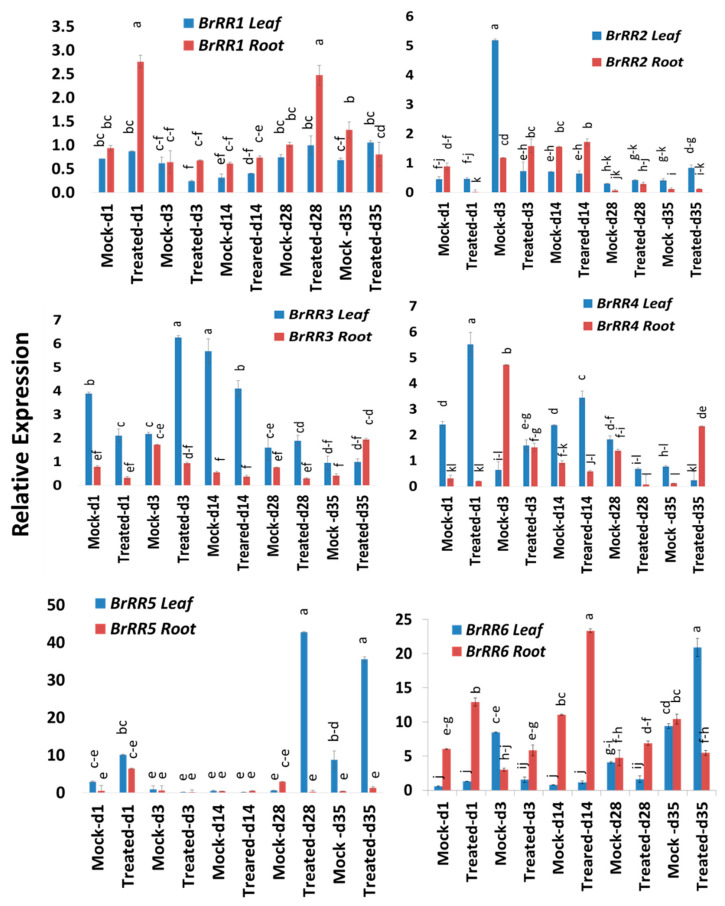
Variation in relative expression levels of *BrRR* cytokinin-related genes in leaf and root samples under different treatment × time-point combinations. Vertical bars compare relative expression levels among leaf vs. root, mock vs. treated samples at five time-points. Each data point represents the average of three samples. Vertical bars represent standard deviation. Values with different letters are significantly different at 5% level of significance according to Tukey’s pairwise comparisons. Gene expression levels in leaves from non-treated plants on Day 0 were set to 1.

**Figure 5:**
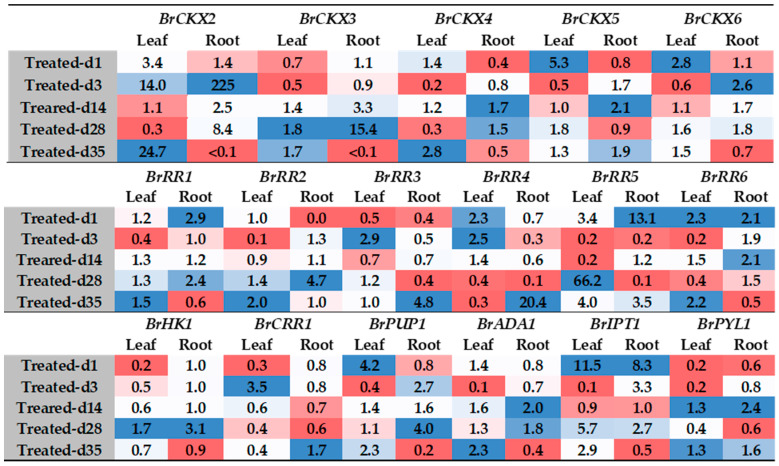
Fold changes in relative expression levels of cytokinin-related genes in Seosan-inoculated plants compared to mock-treated leaf samples at the same time-point. Blue and red colors represent upregulation and downregulation of genes, respectively. *BrCRR1* is involved in protein phosphorylation and *BrPYL1* is a receptor of ABA.

Six response regulator genes, *BrRR1-6,* showed significant variations in time-points, treatment within time-points and sample types within treatment and time-points (Table 2). These response regulators, except *BrRR3* and *BrRR4,* also exhibited significant treatment difference (Table 2). Among the six response regulator genes, *BrRR1* gene exhibited a 2.9- and 2.4-fold increase in expression on Day 1 and Day 28, respectively, in roots compared to mock-treated root samples at the same time-point (Figure 4 and Figure 5; Table 2). *BrRR2* was upregulated 4.7-fold in root tissues on Day 28 in Seosan-inoculated plants compared to mock-treated roots (Figure 4). *BrRR3* gene exhibited a 2.9-fold increase in expression in the leaves of Seosan-inoculated plants compared to mock-treated leaves at the same time-point (Figure 4 and Figure 5). In Seosan-inoculated plants, *BrRR4* expression increased 2.3-fold in leaves on Day 1 and 20.4-fold in roots on Day 35 compared to mock-treated samples (Figure 4). *BrRR5* gene was upregulated 66.2- and 4.0-fold in the leaves of Seosan-inoculated plants on Day 28 and Day 35, respectively, compared to mock-treated leaf samples at the same time-point (Figure 4). *BrRR6* showed a 2.1-, 1.9-, and 2.1-fold increase in expression in Seosan-inoculated root tissues on Day 1, Day 3, and Day 14, respectively, compared to mock-treated root samples at the respective time-points (Figure 5). This gene also showed 2.2-fold higher expression in Seosan-inoculated leaf samples compared to mock-treated samples on Day 35 (Figure 5).

### 2.5. Expression Profiles of Other Cytokinin-Related Genes

**Figure 6:**
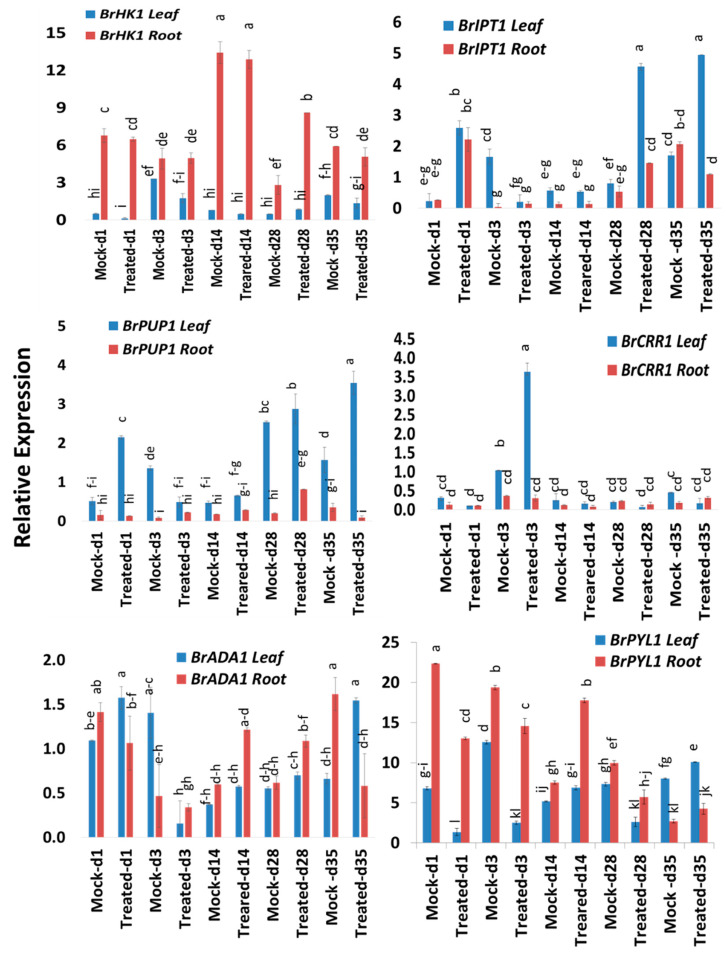
Variation in relative expression levels of cytokinin-related genes *BrHK, BrCRR, BrPUP, BrADA,* and *BrIPT* in leaf and root samples under different treatment × time-point combinations. Vertical bars compare relative expression levels among leaf vs. root, mock vs. treated samples at five time-points. Each data point represents the average of three samples. Vertical bars represent standard deviation. Values with different letters are significantly different at 5% level of significance according to Tukey’s pairwise comparisons. Gene expression levels in leaves from non-treated plants on Day 0 were set to 1.

In addition to investigating the expression patterns of response regulator and cytokinin oxidase genes, we analyzed the expression of five other genes, including histidine kinase, protein kinase, purine permease, transcriptional adaptor, and isopentenyl-transferase genes. At most time-points, the histidine kinase gene *BrHK1* was more highly expressed in root vs. leaf tissue (Figure 6). *BrHK1* gene was expressed at 3.1-fold higher levels in Seosan-inoculated roots on Day 28 compared to mock-treated roots at the same time-point (Figure 5). The protein kinase gene *BrCRR1* was upregulated 3.5-fold in the leaves of Seosan-inoculated plants on Day 3 compared to mock-treated leaf samples (Figure 6).

The purine permease gene *BrPUP1* exhibited a 4.2-fold and 2.3-fold increase in expression on Day 1 and Day 35, respectively, in the leaves of Seosan-inoculated plants compared to mock-treated leaves at the same time-point (Figure 5). The isopentenyl-transferase gene *BrIPT1* exhibited an 11.5-, 5.7-, and 2.9-fold increase in expression on Day 1, Day 28, and Day 35, respectively, in leaf samples of Seosan-inoculated plants compared to mock-treated leaf samples at the same time-point (Figure 5). *BrIPT1* also showed an 8.3- and 2.7-fold increase in expression in Seosan-inoculated root samples on Day 1 and Day 28, respectively, compared to the mock-treated root samples at the same time-point (Figure 5).

The expression levels of transcriptional adapter gene *BrADA1* were quite consistent in all sample types across all time-points. *BrADA1* gene showed 2.3-fold higher expression in leaf samples of Seosan-inoculated plants compared to root samples of mock-treated plants on Day 35 (Figure 6). Finally, *BrPYL1* gene showed a 2.4-fold increase in expression in root tissues on Day 14 in Seosan-inoculated plants compared to the root samples of mock-treated plants (Figure 6).

### 2.6. Expression Profiles of Cytokinin Oxidase Genes

**Figure 7:**
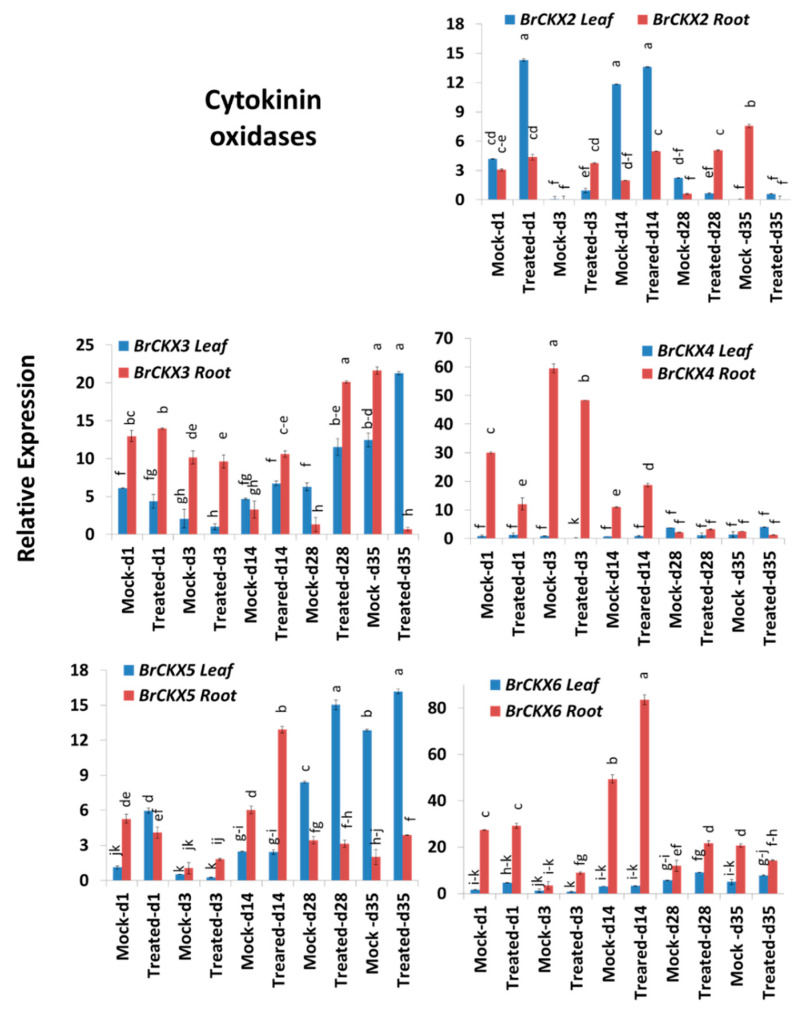
Variation in the relative expression levels of *BrCKX* cytokinin-related genes in leaf and root samples under different treatment × time-point combinations. Vertical bars compare relative expression levels among leaf vs. root, mock vs. treated samples at five time-points. Each data point represents the average of three samples. Vertical bars represent standard deviation. Values with different letters are significantly different at 5% level of significance according to Tukey’s pairwise comparisons. Gene expression levels in leaves from non-treated plants on Day 0 were set to 1.

Cytokinin oxidase genes, *BrCKX2-6,* exhibited significant variations for time-points, treatment, time-points x treatment and sample types within treatment and time-points (Table 2). *BrCKX2* was highly expressed on Day 1, with 3.4-fold higher expression in leaves of Seosan-inoculated plants compared to mock-treated leaves at the same time-point (Figure 5 and Figure 7; Table 2). This gene also showed 225-, 2.5-, and 8.4-fold higher expression in roots on Day 3, Day 14, and Day 27, respectively, compared to mock-treated roots at the same time-point (Figure 5 and Figure 7). *BrCKX3* gene was expressed at 3.3- and 15.4-fold higher levels in Seosan-inoculated roots on Day 14 and Day 28, respectively, compared to mock-treated roots at the same time-point (Figure 5). In general, *BrCKX4* was expressed at higher levels in roots vs. leaves in control plants and in both mock- and Seosan-inoculated plants up to Day 14 compared to leaves (Figure 7). The expression of *BrCKX4* increased 1.7-fold in Seosan-inoculated roots on Day 14 compared to mock-treated roots at the same time-point (Figure 5 and Figure 7). *BrCKX5* was upregulated 5.3-, 1.8-, and 1.3-fold in leaves of Seosan-inoculated plants on Day 1, Day 14, and Day 28 compared to mock-treated leaves at the same time-point (Figure 5 and Figure 7). This gene also showed 2.1-fold increase in expression in Seosan-inoculated roots on Day 14 compared to mock-treated roots at the same time-point (Figure 5). This gene was expressed at significantly (1.7-fold) higher levels in Seosan-inoculated roots on Day 14 compared to mock-treated roots at the same time-point (Figure 7).

## 3. Discussion

The main objective of this study was to assess the transcript levels of cytokinin-related genes in Chinese cabbage inoculated with *P. brassicae* isolate “Seosan” at five different time-points. Cytokinins are one of the major hormone group present in plants those regulated numerous physiological and biochemical processes in plants including growth, development, and metabolism [32,33,34]. We selected “Seosan” isolate because of its high virulence compared to the three other Korean isolates examined.

### 3.1. Expression Level Difference in Leaf vs. Root Tissue

Cytokinins play a crucial role in maintaining root-shoot homeostasis in plants through transduction of nutritional signals [35,36]. In this study, relative expression of several genes including *BrHK1* and *BrIPT1* dramatically changed in root and leaf tissues of both mock and Seosan-inoculated plants at different time-points indicating that hormonal homeostasis within plant is dynamic and that may be changed over time between organs (Figure 3 and Figure 6).

**Figure 8:**
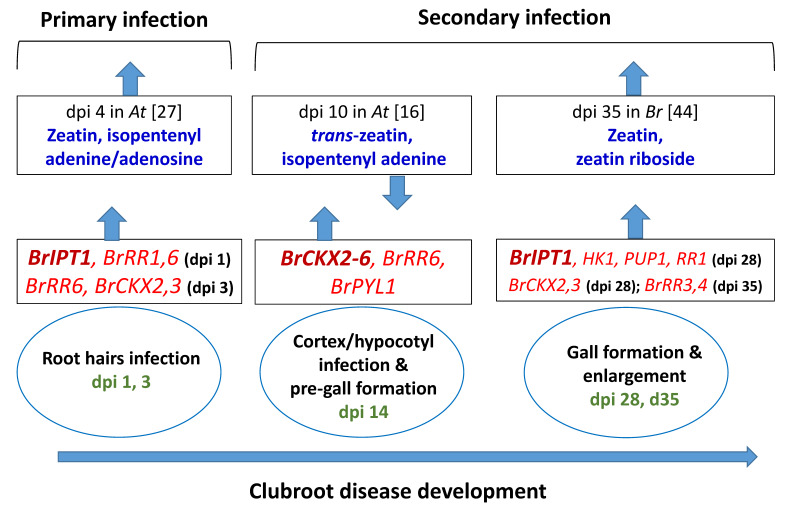
Association between relative expressions of cytokinin biosynthesis, response regulating and degrading genes in this study (red color) and published cytokinin contents at the closer time-points in *Arabidopsis thaliana* and *Brassica rapa* (blue color). dpi, days post inoculation. Green color represents dpi at which samples were collected. Upwards- and downwards-pointing arrows represent upregulation/increase and downregulation/decrease of genes/cytokinin contents, respectively, in clubroot infected plants compared to non-infected plants.

We found that several genes, such as *BrRR1*, *BrRR6*, *BrHK1, BrCKX4,* and *BrCKX6* were expressed at higher levels in root tissues compared to leaf tissues (Figure 3). By contrast, the cytokinin biosynthesis gene *BrIPT1* and response regulators *BrRR3*, *BrRR5,* and *BrPUP1* had lower expression in root tissues indicating that cytokinin levels differ in root vs. leaf tissues considering that expression level of regulating and biosynthesis genes is positively and that of degrading genes is negatively associated with cytokinin biosynthesis (Figure 8, [37]).

### 3.2. Expression Level Difference of BrIPT1 Gene

Isopentenyl-transferase (IPT) is a critically important enzyme for the biosynthesis of cytokinin [38,39,40,41]. This enzyme is vital for cytokinin homeostasis under the infection of *P. brassicae* [42]. In *B. rapa*, Ando et al. [42] found that several *BrIPT* genes, including *BrIPT1,* were induced in the inoculated plants with *P. brassicae* at the first appearance of gall after 20 dpi. In this study a high level of expression of *BrIPT1* gene by 11.5- and 8.3-fold in leaf and root tissues, respectively, on Day 1 after inoculation and a marginal upregulation in inoculated root tissues by 3.3-fold on Day 3 (Figure 5) indicated that primary infection of *P. brassicae* in the root tissues transiently stimulated the expression of this gene. Expression of this gene was repressed on Day 3 and Day 14, during gall initiation (Figure 6). Malinowski et al. [16] reported a comparatively lower expression of *BrIPT1* gene on Day 16 compared to the expression level of *BrIPT3* and *BrIPT5*. A quadruple mutant of IPT- ipt1 ipt3 ipt5 ipt7, measured a very low content of cytokinin in a previous study in *A. thaliana* indicating the importance of *IPT1* gene in cytokinin biosynthesis [43]. This gene again showed 5.7- and 2.7-fold higher expression in leaves and roots of the treated plants, respectively, on Day 28 after inoculation, during gall enlargement, in infected plants indicating its role in gall expansion through induction of cell division (Figure 6). Devos et al. [27] in *Arabidopsis* and Dekhuijzen [44] in *B. rapa* measured significantly higher contents of cytokinin at 4 dpi and 35 dpi, respectively (Figure 8). Increased expression levels of *BrIPT1* gene, in this study, during primary infection of root hairs (on Day 1 and 3) and during gall formation (on Day 28) could be associated with higher biosynthesis of zeatin, isopentenyl adenine, or zeatin riboside in the infected root tissues compared to non-inoculated plants at the adjacent dpi (Figure 8).

### 3.3. Expression Level Difference of BrRR Genes

The BrRR proteins with two receptor (signal transduction) components have a specific histidine kinase to sense environmental changes to mediate response of a cell to environmental homeostasis [45]. The *BrRR1*–*BrRR6* response regulator genes are the Type-A members bearing a short C-terminal extension in addition to the conserved receiver domain [45,46,47]. Exogenous application of trans-zeatin (tZ) transcriptionally upregulated the Type-A gene members [45]. A higher expression level of *BrRR* genes in Chinese cabbage might negatively regulate the expression of *BrHK* and *BrHP* genes instantly [45,48]. Induced expression of *BrRR1* and *BrRR6* during primary infection in roots, coincided with the expression of *BrIPT1* gene. A 20.4-fold higher expression of *BrRR4* gene on Day 35 indicated its vital role during gall enlargement in the infected root tissues as Type-A *RR* genes switch of cytokinin signal (Figure 5 and Figure 8) [16,17,21,26]. In this study only *BrRR4* gene was induced in leaf samples on Day 1, 3, and 14 (Figure 6). The unregulated *BrRR4* gene in leaves may enhance photosynthesis by interacting with phytochrome B after the infection of *P. brassicae* [49].

### 3.4. Expression Level of CKX Genes

The cytokinin oxidase (CKX) enzymes have proven role in irreversible degradation of cytokinins cleaving side-chain in roots and leaves of higher plants [50]. The *CKX* genes in both *A. thaliana* and *B. rapa* differ in subcellular localizations, biochemical properties, and level of expression from spatial and temporal perspectives (Table 1) [51,52]. In transgenic *A. thaliana*, overexpressed plants with six different *CKX* genes showed repressed accumulation of cytokinin that affected root and shoot growth [35]. Exogenous treatment of cytokinin was found to alter the responses of *CKX* genes in *B. napus* [53].

In this study, we analyzed the expression patterns of cytokinin-related genes in leaf and root tissues at different time-points in both non-inoculated and Seosan-inoculated plants. In a previous study, in transgenic *Arabidopsis* plants, the *CKX1*- and *CKX3*-overexpressing plants exhibited increased resistance to *P. brassicae* [26]. We detected notably higher expression of *BrCKX2, BrCKX3*, *BrCKX4*, *BrCKX5,* and *BrCKX6* on Day 14 (before gall formation) in Seosan-treated roots compared to mock-treated root indicating that cytokinin biosynthesis and signaling was repressed on Day 14 (Figure 8) [16,27,28]. Our prediction is supported by the significantly lower accumulation of zeatin and isopentenyl adenine in the clubroot infected root tissues of *Arabidopsis* at 10 dpi in Malinowski et al. [16]. *BrCKX2* and *BrCKX3* were highly induced by 8.4- and 15.4-folds, respectively, on Day 28 when gall appeared but both of these two genes were repressed on Day 35 indicating that cytokinin signaling was enhanced during rapid gall enlargement phase (Figure 5). Previous studies reported that down-regulation of *CKX* genes in *A. thaliana* caused biosynthesis of higher levels of cytokinin upon the infection of *P. brassicae* [22,28].

## 4. Materials and Methods

### 4.1. Preparation of Plant Materials

Seeds of Chinese cabbage (*Brassica rapa* var. pekinensis) cultivar “Bullam-3-ho” were obtained from Woori Seeds, Gwacheon, Gyeonggi, South Korea. The seeds were sown in 50-cell trays containing an artificial soil mixture and placed in a controlled plant culture room (temperature 25 ± 1 °C, relative humidity 65–70%, light intensity 230–250 µmol m^−2^ s^−1^, 16:8 h light-dark cycle) for germination and seedling establishment.

### 4.2. Gall Sample Collection

Four isolates from four different pathotype groups obtained from infected seedlings were previously collected from four different regions of South Korea by Jo et al. [54]. These isolates included Gangneung 1, Daejon, Yeoncheon, and Seosan, belonging to pathotype group 1, 2, 3, and 4, respectively [2].

### 4.3. Spore Preparation from Galls and Infection of Plant Materials

*P. brassicae* spores were extracted from each isolate collected from four different regions of South Korea according to Feng et al. [9] and Laila et al. [4]. The concentration of spores was adjusted to 1 × 10^7^ spores per mL after counting the number of spores in a hemocytometer. Two-week-old plants were inoculated with the four selected isolates via the root cutting method, followed by culture in a controlled environment. Specifically, the plants were inoculated with 6 mL spore suspension (10^7^ spores mL^−1^) for 10‒15 min after cutting the root branches with scissors. The plants were transferred to a 200 cm^3^ pot containing artificial soil in a controlled growth chamber. Mock treatment was performed using 6 mL water.

### 4.4. Disease Scoring Criteria

**Figure 9:**
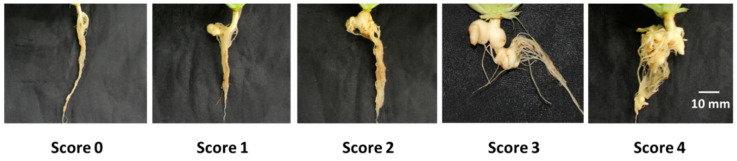
Scoring criteria for clubroot development in Chinese cabbage cultivar “Bullam-3-ho.” Scale of 0–4: 0 = root system without clubs, 1 = one or a few small clubs <5 mm in diameter on lateral roots, 2 = a few medium-sized, separate globular clubs >5 mm in diameter on lateral roots, 3 = medium-sized clubs 5–10 mm in diameter on the main roots, 4 = severe clubbing, with clubs >10 mm in diameter on the lateral and main roots [55].

Inoculated plants were inspected weekly to observe the severity of infection (Figure 9). Each sample was washed with running tap water. The disease severity of clubroot infection was rated on a scale of 0–4 as described by Laila et al. [55] (Figure 9). Disease severity index (DSI) was calculated as the average disease scores of 10 plants (replicates) under each treatment.

### 4.5. Collection of Leaf and Root Samples from Plants Inoculated with Seosan Isolate

For qRT-PCR expression analysis of cytokinin-related genes, 14-day-old seedlings of Chinese cabbage cultivar “Bullam-3-ho” were inoculated with spores of the Seosan isolate (10^7^ spores mL^−1^). Root and leaf samples were harvested from mock and inoculated plants for qRT-PCR analysis at 1, 3, 14, 28 and 35 days after inoculation (DAI). A list of sampling combinations is given in Table 3. Three biological replicates were collected for each sample. During sample collection, any adhering soil residue was carefully removed from the roots using running tap water. The excess water from the roots were then dried between two pieces of filter paper and immediately frozen in liquid nitrogen. The leaf and root samples were stored at −80 °C.

### 4.6. RNA Extraction and cDNA Synthesis

Approximately 100 mg of leaf or root tissue was ground to a powder in liquid nitrogen. Total RNA was extracted from all 22 types of samples using an RNeasy Plant Mini Kit (Qiagen, Hilden, Germany). RNase-free DNase (Qiagen, Hilden, Germany) treatment was performed to remove DNA contamination. The purity of the extracted RNA was determined at a 260/280 nm ratio using a NanoDrop^®^ ND-1000 (Thermo Scientific, Hudson, NH, USA). The cDNA was synthesized from RNA following the manufacturer’s instructions using a Superscript^®^ III First-Strand Synthesis Kit (Invitrogen, CA, USA).

### 4.7. Identification of Cytokinin-Related Genes

Schuller et al. [29] published a list of genes involved in cytokinin metabolism, signaling, and transport in *Arabidopsis thaliana*. In this study, genes were selected based on their high expression level in *Arabidopsis thaliana*. The corresponding *Brassica rapa* orthologs were retrieved from the BRAD database (http://brassicadb.org/brad/; Cheng et al. [56] against each cytokinin-related gene (Table 1, Figure S1). The subcellular localization of each corresponding cytokinin biosynthetic protein was predicted using both UniProt (https://www.uniprot.org/uniprot/) and ProtComp 9.0 from Softberry (http://linux1.softberry.com/berry.phtml). A phylogenetic tree was constructed using Neighbor-joining method in MEGA7.0 software [57]. ProtParam tool [58] was used to determine the properties of the proteins. However, a database research in The Arabidopsis Information Resource (TAIR) (https://www.arabidopsis.org/) identified that At3g55950 (*BrCRR1*, Bra007202) is a protein kinase that is involved in protein phosphorylation. In addition, At5g05440 gene (*BrPYL1*, Bra009113) is an abscisic acid (ABA) binding protein that acts as a receptor of ABA.

### 4.8. qRT-PCR Expression Analysis

We performed qRT-PCR to investigate the relative expression patterns of cytokinin-related genes in 20 samples representing five different time-points, two treatments, and two sample types: leaves and roots. Gene expression levels in leaves from non-treated plants on Day 0 were set to 1. There were two technical replicates for each sample. Cytokinin-related genes-specific primers were used to analyze the gene expression in Seosan-inoculated leaf and root samples via quantitative reverse-transcription PCR (qRT-PCR) (Table 4). The primers were designed based on the coding sequence of each gene using Primer3 software (http://frodo.wi.mit.edu/primer3). The specificity of the primers was checked following Robin et al. [59]. Two different ACTIN genes (GenBank Accession Nos. XM_009147610.2 and FJ969844.1, housekeeping genes) from *B. rapa* were used to normalize the expression levels of cytokinin-related genes. Each 20 μL PCR reaction mixture contained 1 μL template cDNA at 50 ng µL^−1^ concentration, 1 μL each of forward and reverse primers at 10 pmol concentration, 10 μL qPCR BIOSyGreen Mix Lo-ROX (PCR Biosystems, London, UK), and 7 μL ultra-pure double distilled water. The qRT-PCR conditions were as follows: denaturation for 10 min at 95 °C, followed by 40 cycles of amplification at 95 °C for 20 s, 58 °C for 20 s, and 72 °C for 25 s. The quantification cycle value (a measure of fluorescence) was recorded at the end of each cycle. The amplified products were detected and the data were analyzed using a LightCycler96 system (Roche, Mannheim, Germany). The 2^−ΔΔCT^ method was used to calculate the relative expression levels of cytokinin-related genes because equal primer efficiency of the tested genes was used, two stable housekeeping genes were used for standardization, and the selected genes were tested at both control and experimental conditions [60].

### 4.9. Statistical Analysis

Analysis of variance (ANOVA) of relative expression levels of five time-points (dpi), two treatments (mock and Seosan-inoculated), and two sample types (leaf vs. root) was conducted following a nested-ANOVA under general linear model using MINITAB 17 Statistical Software (Minitab Inc., State College, PA, USA) to assess the variation among time-points, treatments, time-point x treatment, and sample type within time-point and treatment. Separate statistical analyses were conducted for disease severity scores and relative expression levels of cytokinin-related genes at two different sample types (leaf vs. root) via one-way ANOVA using MINITAB 17 Statistical Software (Minitab Inc., State College, PA, USA). For pairwise comparisons of means of disease severity scores or relative expression values, Tukey’s pairwise comparisons were performed.

## 5. Conclusions

In this study, the Korean pathotype group 4 isolate Seosan was found to be more virulent than three other isolates, Gangneung1, Daejon, and Yeoncheon, with respect to DSI. The expression patterns of cytokinin-related genes involved in response regulation, biosynthesis, and degradation differed greatly between leaf and root tissues, pointing to their organ-specific expression. Cytokinin biosynthetic gene *BrIPT1* and response regulator genes *BrRR1*, *BrRR4,* and *BrRR6* showed a pattern of upregulation at the beginning of primary infection and during gall expansion. The induced expression of *BrCKX* genes before gall formation indicated their possible role in repression of gall formation. Altered cytokinin contents in root tissues, in the published data, upon the infection of *P. brassicae* temporally coincided with altered expression levels of biosynthetic and catalytic genes of this study. We conclude that the genes *BrIPT1*, *BrRR1*, *BrRR6,* and *BrCKX*s have vital role in clubroot disease development. Multi-genic manipulation of susceptible Chinese cabbage plants with *BrCKX*2-6 genes could be screened for clubroot disease resistance in future. The results of this study improve our current understanding of the possible role of relevant genes in cytokinin signaling during clubroot formation.

## Figures and Tables

**Table 1:** Properties of genes involved in cytokinin metabolism, signaling, and transport in *Brassica rapa.* aa, amino acid; Pi, isoelectric point; MW, molecular weight; ER, endoplasmic reticulum. *BrCRR1* is involved in protein phosphorylation and *BrPYL1* is receptor of ABA.

Gene Name	BRAD ID	Chromosomal Location	Start Codon	Stop Codon	Strand	Gene Identity	(aa)	Pi	MW (kDa)	Subcellular Localization
*BrRR1*	Bra023972	A03	28566478	28568797	−	Two-component response regulator	518	5.92	57.96	Nuclear
*BrHK1*	Bra024849	A06	22745808	22750254	+	Histidine kinase (CRE1/AHK4)	1040	6.21	115.0	Endoplasmic reticulum
*BrRR2*	Bra018084	A06	9832893	9834443	+	Response regulator 5 (ARR5)	179	6.18	20.30	Nuclear
*BrRR2.1*	Bra033773	A01	13811201	13812750	−		180	5.54	20.404	Nuclear
*BrRR3*	Bra036963	Scaffold000123	487890	489100	−	Response regulator 4 (ARR4)	261	4.62	28.525	Nuclear
*BrRR3.1*	Bra031714	A09	36858902	36860112	+		261	4.62	28.525	Nuclear
*BrRR3.2*	Bra019932	A06	3644746	3645865	−		253	4.63	27.682	Nuclear
*BrRR3.3*	Bra018439	A05	8234767	8235847	+		251	4.55	27.317	Nuclear
*BrRR4*	Bra015885	A07	23646646	23647740	−	Response regulator 15 (ARR15)	185	5.94	20.55	Nuclear
*BrRR4.1*	Bra003782	A07	18316602	18317690	+		197	5.06	21.850	Nuclear
*BrRR4.2*	Bra025708	A06	7115317	7116482	−		213	5.54	23.480	Nuclear
*BrPUP1*	Bra010890	A08	16197869	16199402	+	Purine permease (PUP1)	302	8.72	38.77	Nuclear
*BrADA1*	Bra040578	Scaffold000219	46992	49506	−	Transcriptional adaptor (ADA2b)	486	6.51	56.07	Nuclear
*BrADA1.1*	Bra012721	A03	22587864	22590255	−		469	7.53	54.33	Nuclear
*BrIPT1*	Bra001737	A03	18198110	18199443	−	Isopentenyl transferase (IPT8)	327	9.26	37.03	Chloroplast
*BrRR5*	Bra003265	A07	15543847	15545066	+	Response regulator 9 (ARR9)	240	5.07	26.847	Nuclear
*BrRR5.1*	Bra014649	A04	2014110	2015202	−		234	5.20	25.932	Nuclear
*BrRR6*	Bra016526	A08	18109570	18110672	+	Response regulator 7 (ARR7)	207	5.62	23.04	Nuclear
*BrRR6.1*	Bra025708	A06	7115317	7116482	−		213	5.54	23.48	Plasma membrane
*BrCKX2*	Bra036719	A09	5855383	5858908	−	Cytokinin oxidase	502	5.96	55.48	ER
*BrCKX2.1*	Bra040677	Scaffold000232	36707	41121	−		505	5.62	55.60	ER
*BrCKX3*	Bra035640	A02	6020617	6023613	+		518	5.62	58.89	Vacuole/ER
*BrCKX3.1*	Bra002777	A10	7709311	7712297	−		366	5.63	40.97	Vacuole/ER
*BrCKX4*	Bra024135	A03	27423204	27426573	+		524	5.71	57.99	Extracellular
*BrCKX5*	Bra015842	A07	23842777	23845621	−		524	5.62	58.83	Extracellular
*BrCKX6*	Bra007743	A09	32193279	32195072	+		449	7.30	50.498	Extracellular
*BrCRR1*	Bra007202	A09	29452438	29453460	−	Protein kinase family	340	5.71	37.562	Plasma membrane
*BrPYL1*	Bra009113	A10	15189844	15190458	−	ABA binding protein	204	6.02	22.777	Peroxisomal
*BrPYL1.1*	Bra005853	A03	930207	930818	+		203	5.72	22.72	Peroxisomal
*BrPYL11.2*	Bra028758	A02	798022	798519	+		165	5.52	18.37	Peroxisomal

**Table 2:** Sources of variation, degrees of freedom (df), mean squares (MS), F statistic, *p* value for relative expression levels of 17 cytokinin-biosynthesis related genes in leaf and root samples at five different time-points (1, 3, 14, 28, and 35 days after inoculation) under two different treatments (mock and inoculation with Seosan-isolate of *P. brassicae*) in Chinese cabbage cultivar “Bullam-3-ho.”

Sources of Variation	df	Genes	MS	F Statistic	*p* Value	Genes	MS	F Statistic	*p* Value
Time-point (Tm)	4	*BrRR1*	1.84	253.7	<0.01	*BrADA1*	1.05	62.5	<0.01
Treatment (Tr)	1		1.74	239.4	<0.01		0.0003	0.02	0.891
Tm x Tr	4		0.88	121.1	<0.01		0.559	33.06	<0.01
Leaf vs. Root (Tm Tr)	10		1.01	139	<0.01		0.56	33.13	<0.01
Error	40		0.033				0.016		
Time-point	4	*BrRR2*	7.64	1305.2	<0.01	*BrIPT1*	7.52	671.2	<0.01
Treatment	1		2.46	420.9	<0.01		12.2	1089.8	<0.01
Tm x Tr	4		2.67	457.9	<0.01		5.58	498.3	<0.01
Leaf vs. Root (Tm Tr)	10		2.97	507.1	<0.01		5.63	502.3	<0.01
Error	40		0.055				0.0112		
Time-point	4	*BrRR3*	7.98	241.5	<0.01	*BrPYL1*	85.1	642.1	<0.01
Treatment	1		0.068	2.07	0.158		79.3	598.1	<0.01
Tm x Tr	4		4.04	122.4	<0.01		106.4	802.7	<0.01
Leaf vs. Root (Tm Tr)	10		12.8	389.9	<0.01		115.9	873.8	<0.01
Error	40		0.033				0.133		
Time-point	4	*BrRR4*	4.48	242.5	<0.01	*BrCKX2*	114.1	562.9	<0.01
Treatment	1		0.073	3.95	0.054		41.4	204.5	<0.01
Tm x Tr	4		4.41	238.6	<0.01		32.8	161.9	<0.01
Leaf vs. Root (Tm Tr)	10		9.74	526.8	<0.01		53.8	265.8	<0.01
Error	40		0.018				0.203		
Time-point	4	*BrRR5*	376.1	191.9	<0.01	*BrCKX3*	131.6	586.8	<0.01
Treatment	1		925.2	472.2	<0.01		53.9	240.4	<0.01
Tm x Tr	4		235.2	120.1	<0.01		139.8	623.2	<0.01
Leaf vs. Root (Tm Tr)	10		461.6	235.6	<0.01		135.2	602.5	<0.01
Error	40		1.959				0.224		
Time-point	4	*BrRR6*	121.8	479.8	<0.01	*BrCKX4*	1248.8	2545.6	<0.01
Treatment	1		75.4	297.0	<0.01		70.6	144.1	<0.01
Tm x Tr	4		33.4	131.5	<0.01		78.8	160.6	<0.01
Leaf vs. Root (Tm Tr)	10		161.9	637.8	<0.01		1074.3	2189.9	<0.01
Error	40		0.254				0.49		
Time-point	4	*BrHK1*	32.04	351.1	<0.01	*BrCKX5*	112,4	2488.7	<0.01
Treatment	1		0.05	0.56	0.458		75.6	1675.7	<0.01
Tm x Tr	4		12.55	137.6	<0.01		4.89	108.4	<0.01
Leaf vs. Root (Tm Tr)	10		72.8	798.7	<0.01		87.3	1933.2	<0.01
Error	40		0.09				0.045		
Time-point	4	*BrCRR1*	3.19	968.4	<0.01	*BrCKX6*	1618.7	2676.6	<0.01
Treatment	1		0.47	145.3	<0.01		431.9	714.2	<0.01
Tm x Tr	4		1.11	336.8	<0.01		157.1	259.8	<0.01
Leaf vs. Root (Tm Tr)	10		1.74	530.6	<0.01		1558.9	2577.8	<0.01
Error	40		0.003				0.60		
Time-point	4	*BrPUP1*	3.43	354.4	<0.01				
Treatment	1		2.21	227.4	<0.01				
Tm x Tr	4		0.769	79.2	<0.01				
Leaf vs. Root (Tm Tr)	10		4.38	451.8	<0.01				
Error	40		0.009						

**Table 3:** Twenty samples collected from Seosan-inoculated and non-inoculated plants at five different time-points under two different treatments.

Sample ID	Treatment	Time-Point	Sample Type
1	Mock	Day 1	Leaf
2	Mock	Day 1	Root
3	Seosan-inoculated	Day 1	Leaf
4	Seosan-inoculated	Day 1	Root
5	Mock	Day 3	Leaf
6	Mock	Day 3	Root
7	Seosan-inoculated	Day 3	Leaf
8	Seosan-inoculated	Day 3	Root
9	Mock	Day 14	Leaf
10	Mock	Day 14	Root
11	Seosan-inoculated	Day 14	Leaf
12	Seosan-inoculated	Day 14	Root
13	Mock	Day 28	Leaf
14	Mock	Day 28	Root
15	Seosan-inoculated	Day 28	Leaf
16	Seosan-inoculated	Day 28	Root
17	Mock	Day 35	Leaf
18	Mock	Day 35	Root
19	Seosan-inoculated	Day 35	Leaf
20	Seosan-inoculated	Day 35	Root

**Table 4:** List of primer sequences used for qRT-PCR of cytokinin-related genes. *BrCRR1* is involved in protein phosphorylation and *BrPYL1* is a receptor of ABA.

Arabidopsis Homolog (Accession Number)	Gene Name	BRAD ID	cDNA Size (bp)	Primer Forward (F) and Reverse(R)	Product Size (bp)
At4g31920	*BrRR1*	Bra023972	1557	F:AGCTCAAGAACATATGGCAA	191
				R:TGGATCATCGTTCTCATTCC	
At2g01830	*BrHK1*	Bra024849	3123	F:AGCTCTGAAGAAGTTTGGAG	200
				R:GTAAATGCCATTCCAGCTTC	
At3g48100	*BrRR2*	Bra018084	540	F:TACTCAGAGTCTCTTCGTGT	171
				R:CTTCTTGAGTAGTTCATATC	
At1g10470	*BrRR3*	Bra036963	786	F:GTTCAGAGATGATGAGGGTC	208
				R:GCAGATGCTTTCTCGTTGTC	
At1g74890	*BrRR4*	Bra015885	558	F:TCTACCTCGGAGTTACATGT	161
				R:CCAGAAGATCCTTTGTCTCC	
At3g55950	*BrCRR1*	Bra007202	1023	F:GATTACTATGGGTGAGCTGG	167
				R:CTCTCTCCAAATTTCCGACA	
At1g28230	*BrPUP1*	Bra010890	1059	F:TAGCTTTCAACGCTCTCTTT	159
				R:CAACCACATACTCCTTGTGA	
At4g16420	*BrADA1*	Bra040578	1461	F:ATGAAATGCTTCTCCTGGAG	178
				R:TTCTTGTTCTTCCCTGCTAC	
At3g19160	*BrIPT1*	Bra001737	984	F:TTCTGAGCTGAGGTACGATT	194
				R:ACTCCGGTACTCCTATAGCC	
At3g57040	*BrRR5*	Bra003265	723	F:GGCATGACTGGTTATGATTT	237
				R:AAGAGATTGAAGCACCTTCA	
At1g19050	*BrRR6*	Bra016526	624	F:TTAGTTCGCCTGACCTACAT	163
				R:TCAGAATCTCCTGTGTTTCC	
At5g05440	*BrPYL1*	Bra009113	615	F:GAGAGGCTCGAGATCCTGGAC	237
				R:ACAGCTTCTAGCCAGAGACTG	
At2g19500	*BrCKX2*	Bra036719	1509	F:TTAGTGAAGAGCCACGGTAT	210
				R:CCATAAACCCAAAGATCTGA	
At5g56970	*BrCKX3*	Bra035640	1557	F:GGGAGAGCTAAACCTGAAAT	238
				R:TAAGAACCCTACCGCATAAA	
At4g29740	*BrCKX4*	Bra024135	1575	F:GCTGGTGGATCACATAACTT	170
				R:ATTTTCCTCCTCGAGAAATC	
At1g75450	*BrCKX5*	Bra015842	1575	F:GTATCTTTCCGTTGGTGGTA	200
				R:CTCGTGTAATGATCCCAAAT	
At3g63440	*BrCKX6*	Bra007743	1350	F:GGTTATCCTTCACACCAGAA	225
				R:AGACATGTACCCTGTCCAAG	
XM_009147610.2	*ACTIN1*		1371	F: CAACCAATCGTCTGTGACAA	105
				R: ATGTCTTGGCCTACCAACAA	
FJ969844.1	*ACTIN2*		491	F: AATGGTACCGGAATGGTCAA	119
				R: TCCTTCTGGTTCATCCCAAC	

## Supplementary Materials

The following are available online at https://www.mdpi.com/1422-0067/21/11/3896/s1, Figure S1. Phylogenetic tree showing association between cytokinin biosynthesis genes (black) in *Arabidopsis thaliana* and orthologues in *Brassica rapa*. *BrCRR1* is involved in protein phosphorylation and *BrPYL1* is a receptor of ABA. Table S1. Variation in relative expression of cytokinin regulating, synthesizing and degrading genes in root and leaf samples showing non-significant variation between leaves and roots. Each data is the average (±sd) of both inoculated and non-inoculated samples at five different time points. *BrCRR1* is involved in protein phosphorylation and *BrPYL1* is a receptor of ABA.
