# Supplementary material for: Expression and Role of Response Regulating, Biosynthetic and Degrading Genes for Cytokinin Signaling during Clubroot Disease Development
Source: Int J Mol Sci. 2020 May 29;21(11):3896. doi: 10.3390/ijms21113896 (PMC7312684; doi:10.3390/ijms21113896)
Supplement: Supplementary file 1 [file ijms-21-03896-s001.pdf]

Expression and Role of Response Regulating, Biosynthetic and Degrading Genes for Cytokinin Signaling during Clubroot Disease Development

Rawnak Laila, Arif Hasan Khan Robin, Jong-In Park, Gopal Saha, Hoy-Teak Kim, Md. Abdul Kayum and Ill-Sup Nou

Supplementary Data

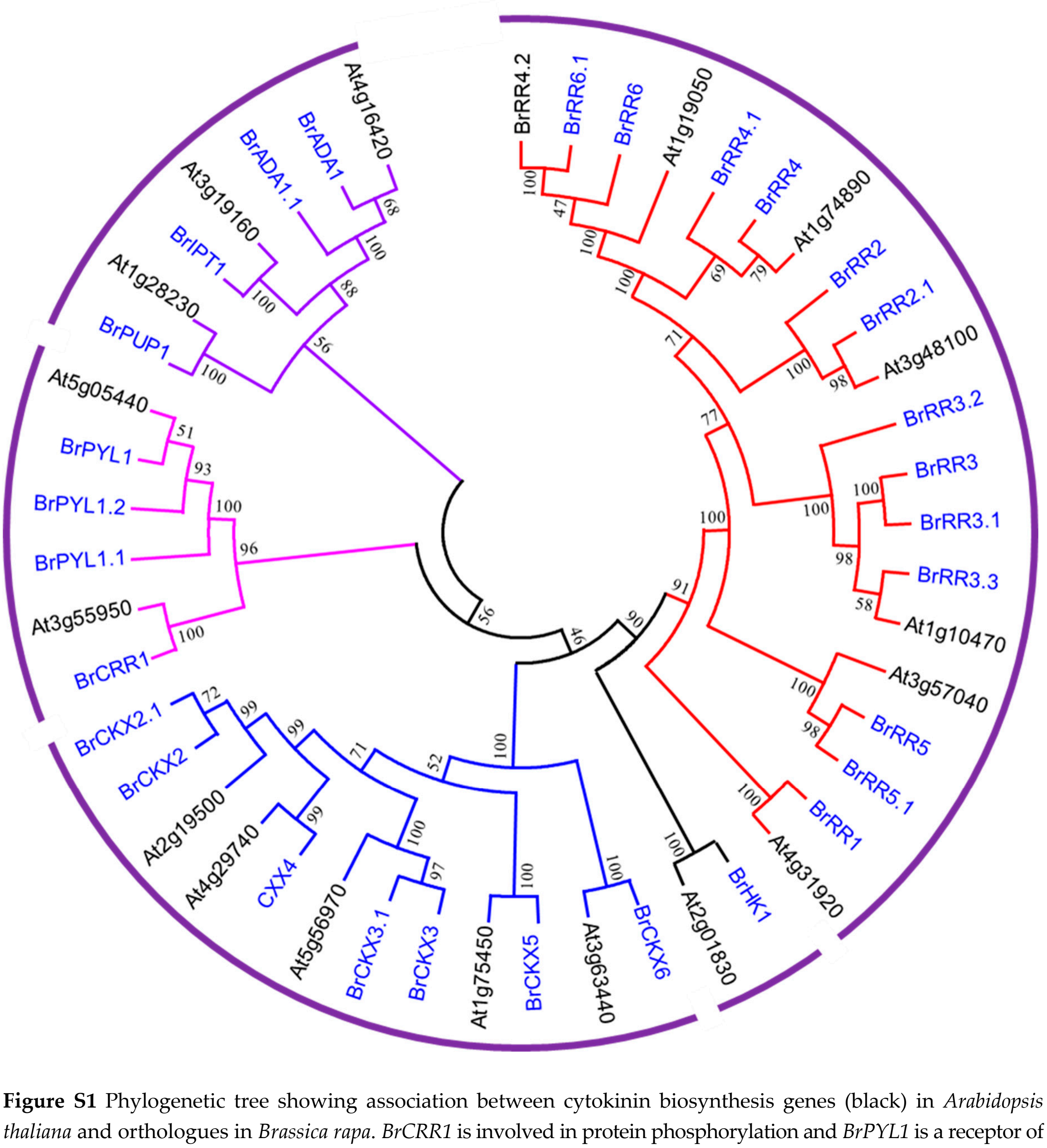

Figure S1 Phylogenetic tree showing association between cytokinin biosynthesis genes (black) in *Arabidopsis thaliana* and orthologues in *Brassica rapa*. *BrCRR1* is involved in protein phosphorylation and *BrPYL1* is a receptor of ABA.

Table S1. Variation in relative expression of cytokinin regulating, synthesizing and degrading genes in root and leaf samples showing non-significant variation between leaves and roots. Each data is the average (±sd) of both inoculated and non-inoculated samples at five different time points. *BrCRR1* is involved in protein phosphorylation and *BrPYL1* is a receptor of ABA.

Variable	*BrRR2*	*BrRR4*	*BrADA1*	*BrPYL1*	*BrCRR1*	*BrCKX2*	*BrCKX3*	*BrCKX5*
Leaf	1.02±1.4	1.94±1.5	0.86±0.5	6.33±3.4	0.61±1.1	4.85±5.6	7.65±5.8	6.53±5.9
Root	0.75±0.68	1.21±1.4	0.89±0.43	11.7±6.4	0.19±0.2	3.14±2.4	10.4±6.9	4.35±3.3
P value	0.36	0.06	0.76	0.07	0.08	0.14	0.10	0.09
